# Supplementary material for: Preoperative inflammation and nutrition-based comprehensive biomarker for predicting prognosis in resectable colorectal cancer
Source: Front Oncol. 2023 Nov 22;13:1279487. doi: 10.3389/fonc.2023.1279487 (PMC10699855; doi:10.3389/fonc.2023.1279487)
Supplement: Supplementary file 1 [file Presentation_1.pdf]

In this study, we examined various blood indices that impact the prognosis of colorectal cancer (CRC) patients. These indices encompassed neutrophil count (NEUT), lymphocyte count(LY), monocyte count (MONO), along with several calculated indices: the systemic immune-inflammation index (SII), defined as (platelet count × neutrophil count) / lymphocyte count, the systemic inflammation response index (SIRI), defined as (neutrophil count × monocyte count) / lymphocyte count, Onodera's Prognostic Nutritional Index (OPNI), defined as (serum albumin + 5 × lymphocyte count), neutrophil-to-lymphocyte ratio (NLR), platelet-to-lymphocyte ratio (PLR), and monocyte-to-lymphocyte ratio (MLR). A dimensionality reduction process was performed using R software. By employing the LASSO regression program, we quantified the impact of these nine indicators on the endpoint event. The coefficients are depicted in **Fig.S1**. Notably, the coefficient for neutrophil count was -0.3295, the coefficient for SIRI was -7.2153, and the coefficient for OPNI was 0.9858. The remaining indicators were excluded due to their minimal impact on the comprehensive assessment's endpoint event, resulting in no coefficient output. Neutrophil count were excluded because of its relatively low coefficient, and the assessment of neutrophil count was already incorporated in the calculation of SIRI. Ultimately, we introduced the novel prognostic indicator PSI, defined as (0.9858 × OPNI) - (7.2153 × SIRI).

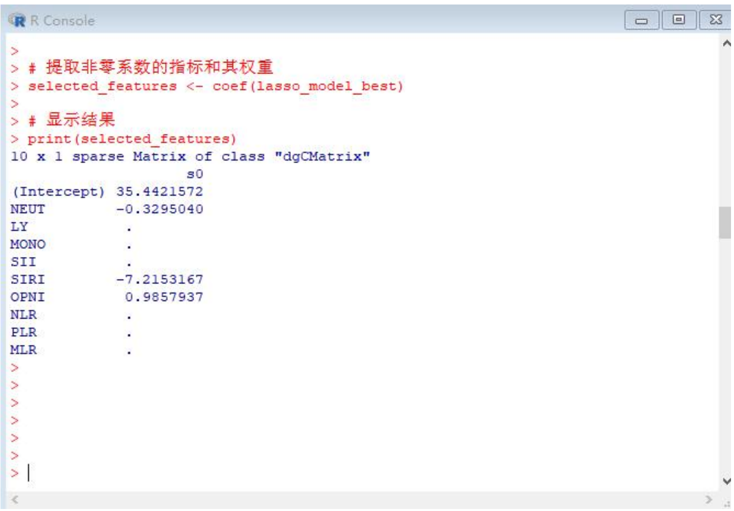

Fig.S1 Coefficients of Nine Prognostic Indicators in resectable CRC.

Abbreviations : *NEUT* neutrophil count, *LY* lymphocyte count, *MONO* monocyte count, *SII* systemic immune-inflammation index, *SIRI* systemic inflammation response index, *OPNI* Onodera's Prognostic Nutritional Index, *NLR* neutrophil-to-lymphocyte ratio, *PLR* platelet-to-lymphocyte ratio, *MLR* monocyte-to-lymphocyte ratio.
